# Mechanisms of Pulmonary Hypertension in Acute Respiratory Distress Syndrome (ARDS)

**DOI:** 10.3389/fmolb.2020.624093

**Published:** 2021-01-18

**Authors:** Lucy Revercomb, Ankit Hanmandlu, Nancy Wareing, Bindu Akkanti, Harry Karmouty-Quintana

**Affiliations:** ^1^Department of BioSciences, Rice University, Houston, TX, United States; ^2^Department of Biochemistry and Molecular Biology, McGovern Medical School, University of Texas Health Science Center at Houston, Houston, TX, United States; ^3^Divisions of Critical Care, Pulmonary and Sleep Medicine, McGovern Medical School, University of Texas Health Science Center at Houston, Houston, TX, United States

**Keywords:** hyaluronan, renin angiotensin system, interleukin-6 (IL-6), vascular dysfunction and inflammation, acute lung injury, vasoconstriction, vascular remodelling

## Abstract

**Background:** Acute respiratory distress syndrome (ARDS) is a severe and often fatal disease. The causes that lead to ARDS are multiple and include inhalation of salt water, smoke particles, or as a result of damage caused by respiratory viruses. ARDS can also arise due to systemic complications such as blood transfusions, sepsis, or pancreatitis. Unfortunately, despite a high mortality rate of 40%, there are limited treatment options available for ARDS outside of last resort options such as mechanical ventilation and extracorporeal support strategies.

**Aim of review:** A complication of ARDS is the development of pulmonary hypertension (PH); however, the mechanisms that lead to PH in ARDS are not fully understood. In this review, we summarize the known mechanisms that promote PH in ARDS.

**Key scientific concepts of review:** (1) Provide an overview of acute respiratory distress syndrome; (2) delineate the mechanisms that contribute to the development of PH in ARDS; (3) address the implications of PH in the setting of coronavirus disease 2019 (COVID-19).

## Introduction

Acute respiratory distress syndrome (ARDS) is a severe form of lung respiratory failure characterized by diffuse alveolar damage, inflammation, and acute onset of hypoxemia not explained by cardiac failure. Diagnostic criteria for ARDS include (1) presentation within 1 week of a known clinical insult, new, or worsening respiratory symptoms; (2) bilateral opacities consistent with pulmonary edema on chest imaging; (3) respiratory failure not fully explained by cardiac failure or fluid overload; and (4) physiologic values of arterial oxygen partial pressure to fractional inspired oxygen (PaO_2_/FIO_2_) ≤ 300 mmHg and positive end-expiratory pressure (PEEP) ≥5 cm H_2_O (The ARDS Definition Task Force, 2012).

Several chronic disorders have been associated with the development of ARDS; however, the majority of ARDS develops from a pulmonary or non-pulmonary infection (Matthay and Zemans, 2011). ARDS can be triggered by many pathogenic conditions including sepsis, pancreatitis, and respiratory viruses such as the H5N1 avian influenza virus, the 1918 influenza pandemic, the severe acute respiratory syndrome-coronavirus (SARS-CoV), and most recently SARS-CoV-2 that causes coronavirus disease 2019 (COVID-19) (Imai et al., 2010; Rezoagli et al., 2017).

The pathogenesis of ARDS is typically divided into the acute, subacute, and chronic phases (Matthay and Zemans, 2011). The acute phase marks the first 1–6 days and is characterized by interstitial and alveolar edema, endothelial and epithelial injury, and the accumulation of neutrophils, macrophages, and red blood cells in the alveoli (Matthay and Zemans, 2011). The subacute phase marks the next 7–14 days and is characterized by some reabsorption of the edema, evidence of repair attempts by alveolar epithelial type II (AE2) cells, infiltration of fibroblasts, and collagen deposition (Matthay and Zemans, 2011). After 14 days, ARDS is considered to be in the chronic phase and there is often resolution of the acute neutrophilic infiltrate, accumulation of mononuclear cells and alveolar macrophages in the alveoli, and more fibrosis resulting from the alveolar epithelial repair (Matthay and Zemans, 2011).

ARDS is observed in 10–15% of admitted patients in intensive care units and more than 20% of patients undergoing invasive mechanical ventilation (Frutos-Vivar et al., 2004). In addition to this high prevalence and despite best measures, ARDS has a high mortality rate of up to 45% (Bellani et al., 2016). Treatment of ARDS is complex, involving ventilatory and non-ventilatory management strategies including conservative fluid management, low tidal volume ventilation, prone positioning, inhaled vasodilator therapy and in refractory cases, extra corporeal membrane oxygenation (Peek et al., 2009; Calcaianu et al., 2017).

ARDS is frequently complicated by pulmonary hypertension (PH), a pathologic condition involving a progressive increase in pulmonary vascular resistance (PVR) leading to right ventricular dysfunction (RVD) that ultimately results in RV failure (RVF) (Simonneau et al., 2019). PH is clinically defined as mean pulmonary arterial pressure (mPAP) > 20 mmHg measured by right heart catheterization (Simonneau et al., 2019). PH in the setting of ARDS falls under group 3 PH due to the presence of hypoxia and lung injury (Poor et al., 2012).

Potential underlying mechanisms of PH in ARDS include vessel obliteration, pulmonary vasoconstriction, and microthrombosis due to hypoxia, hypercapnia, and an imbalance in vasoactive mediators (Price and Wort, 2017). As an inflammatory condition, ARDS is characterized by endothelial cell injury and dysfunction (Price and Wort, 2017). Early in the pathogenesis of ARDS, thromboembolism, pulmonary vasoconstriction, and interstitial edema contribute to the development of PH by collectively elevating PVR (Moloney and Evans, 2003). Damage to the endothelium of the lung results in the accumulation and activation of neutrophils in the lung microvasculature, leading to degranulation and the release of toxic mediators including proteases, reactive oxygen species, proinflammatory cytokines, and procoagulant molecules, which promote vasoconstriction and an increase in PVR (Matthay and Zemans, 2011). The release of tissue factor (TF), an activator of the extrinsic clotting cascade, by endothelial cells elevates PVR through the local formation of microthrombi which obstruct blood flow (Ryan et al., 2014). Vascular remodeling and proliferation of smooth muscle cells through the release of endothelin-1 (ET-1) in the subacute and chronic phases of ARDS occludes the pulmonary vasculature, further increasing PVR and the accumulation of interstitial edema, contributing to the development of PH (Moloney and Evans, 2003). In addition, fibrocellular obliteration of the microvasculature in late phase ARDS disrupts blood flow and helps sustain the elevated PVR that leads to PH (Ryan et al., 2014).

Many of these mediators that are triggered after lung damage in ARDS are also central in the pathophysiology of PH, where injury to endothelial cells and increased levels of ET-1 and TF also contribute to increased vascular tone and remodeling in PH (Moloney and Evans, 2003; Tamosiuniene et al., 2011; Antoniak et al., 2014).

It has been proposed that the increase in inflammation characteristic of ARDS and secondary to ventilator-induced lung injury prompts the pulmonary vascular injury observed in PH (Meduri et al., 2009; Price and Wort, 2017). The incidence of RVD associated with ARDS has declined with the adoption of improvements in mechanical ventilation which lessen the intrathoracic airway pressure in ARDS patients (Vieillard-Baron et al., 2001). The ARDSnet trial has shown significant mortality benefit, with improvement of low tidal volume ventilation for RVD, and improvement of acute cor pulmonale, in severe cases of ARDS (Brower et al., 2000).

Despite advances in the management of ARDS, prevalence of PH in ARDS remains high. A 92% prevalence of PH was reported by Beiderlinden et al. (2006). Ñamendys-Silva et al. (2014), who used a strict selection criteria excluding any patients with clinical conditions that may have predisposed them to PH before the onset of ARDS, reported a prevalence of 46.6%. Villar et al. (1989) reported a similar prevalence of 54%. These findings are summarized in Table 1 where mean pulmonary arterial pressure (mPAP) values determined by right-heart catheterization values ranged from 29 to 36 mmHg in patients with PH. The prevalence of acute cor pulmonale, the severest form of RV dysfunction in the setting of ARDS, is still reported to be as high as 20% (Dessap et al., 2016).

**Table 1:** Prevalence of PH in ARDS.

**mPAP (mmHg)**					
**All patients**	**Without PH**	**PH**	**Method**	**Prevalence (%)**	**References**
35.4 ± 8.8			Right Heart Catheterization	92.2	Beiderlinden et al., 2006^*^
27.07 ± 10.29	19.19 ± 3.78	36.07 ± 7.50	Right Heart Catheterization	46.6	Ñamendys-Silva et al., 2014
	15 ± 3	29 ± 6	Right Heart Catheterization	54	Villar et al., 1989

* *individual mPAP values for patients with and without PH not provided*.

ARDS is a complex disease with multifactorial consequences; as such, in addition to elevated mPAP, other parameters have been strongly associated with mortality such as partial pressure of carbon dioxide (PaCO_2_), partial pressure of oxygen (PaO_2_), or PaO_2_/fraction of inspired oxygen (FiO_2_). Additionally, pulmonary vascular dysfunction, as measured by an increase in transpulmonary pressure gradient and PVR, was associated with increased mortality in ARDS patients (Monchi et al., 1998; Meduri et al., 2009; Bull et al., 2010; Calcaianu et al., 2017). In addition, the composite marker diastolic pulmonary gradient >7 mmHg and PVR >3 Wood Units (WU) seemed to provide a better description of hemodynamic and respiratory dysfunction than other measurements, correlating with a more severe illness and worse patient outcomes (Calcaianu et al., 2017). These findings are summarized in Table 2 and underscore disruptions in the lung vasculature in ARDS as potential disease-amplifying effects that are not fully understood. Although a study showed no correlation between RVF and mortality in ARDS (Osman et al., 2009), other studies have reported an association between RVD and increased morbidity and mortality in ARDS (Monchi et al., 1998; Bull et al., 2010). Further, due to the effects on organ failure in severe ARDS, the severity of organ failure at admission is an important predictor of mortality (Ñamendys-Silva et al., 2014; Bellani et al., 2016).

**Table 2:** Factors associated with mortality of PH in ARDS.

**Variable**	**Survivors**	**Non-survivors**	***P-*value**	**References**
PaO_2_ (mmHg)	75.95 ± 32.96	74.44 ± 16.47	0.888	Ñamendys-Silva et al., 2014
PaO_2_/FiO_2_ (mmHg)	144.64 ± 47.84	135.64 ± 44.81	0.616	
PaCO_2_ (mmHg)	34.51 ± 5.15	40.59 ± 8.44	<0.001	
mPAP (mmHg)	28.32 ± 10.98	24.91 ± 9.08		
PaO_2_/FiO_2_	112 ± 39	93 ± 31	<0.05	Osman et al., 2009
PaCO_2_	43 ± 7	48 ± 16	<0.05	
mPAP	27 ± 6	28 ± 8	<0.01	
PaO_2_	88.7 ± 40.1	78.1 ± 38.5	0.002	Squara et al., 1998
PaO_2_/FiO_2_	149 ± 74	120 ± 59	0.0001	
PaCO_2_	37.1 ± 8.2	38.8 ± 9.0	0.02	
mPAP	25.1 ± 7.3	26.8 ± 9.0	0.04	
mPAP	31.3 ± 8.3	32.4 ± 8.3	0.18	Bull et al., 2010
DPG	14.3 (11.3–18.3)	15.7 (12.3–22.3)	0.02	
PVRi	299.9 (199.4–416.1)	326.4 (206.4–518.7)	0.02	
Δ PaO_2_/FiO_2_ <0 (mmHg)			0.007	Calcaianu et al., 2017

Although PH is a frequent complication of ARDS, there is not a definitive correlation between the presence of PH and the severity and mortality of ARDS. Regardless, in instances of ARDS with or without PH, the mortality rate is very high. The toll of this mortality rate, and accordingly the need for novel therapies, has been demonstrated by the impact and death resulting from the pandemic of COVID-19 where pulmonary vascular abnormalities are present (Potus et al., 2020). A more comprehensive understanding of the role and mechanism of PH in ARDS is needed to improve therapies. In this review, we discuss the central pathways that contribute to PH in ARDS.

## Experimental Methodology

We reviewed the current and past literature on ARDS to identify potential mechanisms that promote PH in ARDS.

## Results

### The Renin–Angiotensin System

The renin–angiotensin system (RAS) is a primary cardiovascular regulatory system responsible for the regulation of blood pressure and electrolyte balance. RAS dysfunction has been proposed as a key pathogenic mechanism of inflammatory lung disease, including ARDS (Imai et al., 2010; Jia, 2016). Genetic variants in the RAS pathway have recently been implicated in the onset and severity of ARDS, specifically angiotensin-converting enzyme (ACE), and its derivatives (Imai et al., 2010). ACE is a peptidase that regulates RAS by cleaving angiotensin I (AngI) to generate angiotensin II (AngII). AngII subsequently binds to AngII receptor type 1 (AT1R) and AngII receptor type II (AT2R) to regulate RAS (Imai et al., 2010). It is primarily through AT1R that AngII causes growth and proliferation of pulmonary artery smooth muscle cells (PASMCs) correlated with pulmonary vascular remodeling (Morrell et al., 1999). This is attributed to AngII-mediated increases in expression of pro-inflammatory genes including interleukin-10 (IL-10), interleukin-6 (IL-6), tumor necrosis factor (TNF-α), and intracellular adhesion molecule-1 (ICAM-1) in fibroblasts and smooth muscle cells (Xianwei et al., 2012). Subsequent vascular remodeling and fibrotic changes contribute to the pathogenesis and progression of pulmonary fibrosis and PH (Kuba et al., 2006). Further, activation of AT1R by AngII promotes vasoconstriction (Iwai and Horiuchi, 2009), adding to the deleterious properties of AngII.

Angiotensin-converting enzyme 2 (ACE2) is an ACE homolog which regulates RAS through counterbalancing ACE activity. This homolog was termed ACE2 after it was identified and cloned from cDNA libraries by two independent groups (Donoghue et al., 2000; Tipnis et al., 2000). While ACE and ACE2 share 41.8% sequence identity, ACE2 negatively regulates RAS, opposing the role of ACE (Donoghue et al., 2000; Tipnis et al., 2000; Imai et al., 2010). ACE2 cleaves a single residue from AngI to yield angiotensin1–9 (Ang1–9) and removes a single residue from AngII to yield angiotensin1–7 (Ang1–7) (Donoghue et al., 2000; Tipnis et al., 2000), which downregulates AT1R-mediated actions by reducing AngII levels (Ferreira and Santos, 2005). In addition, Ang1–7 and Ang1–9 oppose AT1R through anti-inflammatory and antifibrotic actions, by binding the Mas receptor (MasR) and AT2R, respectively (Kreutz et al., 2020; South et al., 2020). In the pathogenesis of ARDS, ACE, AngII, and AT1R promote inflammation and PH, while ACE2, Ang1–7, and Ang1–9 serve to protect against ARDS and PH (Imai et al., 2005).

There is a polymorphism of ACE defined by the absence (deletion, D) or presence (insertion, I) of a 287-bp repeat in the coding sequence of intron 16 (Rigat et al., 1990). The human ACE D allele results in increased activity of ACE demonstrated by an increase in serum ACE levels. This polymorphism accounts for 47% of the variance in plasma ACE activity (Rigat et al., 1990). There also exists a pronounced correlation between the D allele and the development of ARDS, suggesting the role of RAS activation early in disease (Marshall et al., 2002). In addition, the ACE D/D allele correlates with increased mortality in ARDS especially in comparison with the ACE I/I allele which shows increased survival rate, marking the I/D polymorphism as a significant prognostic factor for ARDS outcome (Jerng et al., 2006). Thus, it is conceivable that the D allele may worsen PH in ARDS.

ACE2 expression has been located on lung alveolar epithelial cells (AECs) and enterocytes of the small intestine, and additionally on arterial smooth muscle cells and arterial and venous endothelial cells in all organs (Hamming et al., 2004). These areas of expression indicate an abundant presence of ACE2 in the lung and small intestine epithelia (Hamming et al., 2004). In addition, increased expression of ACE has been found in the muscularized intra-acinar pulmonary arteries of patients with PH, supporting the proposed role of ACE in PH (Orte et al., 2000), through the upregulation of AngII.

Many experiments have evaluated and found supporting evidence of the role of ACE in PH promoting inflammation and vascular dysfunction, with the activity of ACE2 serving as an important counterbalance. Experimental PH was associated with elevated ACE expression in the endothelial layer of small and elastic pulmonary arteries (Morrell et al., 1995; Orte et al., 2000; Schuster et al., 2012). While a significant decrease in ACE2 activity was observed in human pulmonary arterial hypertension (PAH) patients (Hemnes et al., 2018), ACE2 knockout mice display worsened lung function, increased vascular permeability, enhanced lung edema, and neutrophil accumulation, attributed to a downregulation of ACE2 (Imai et al., 2005). Later treatment of the ACE2-deficient mice with catalytically active recombinant ACE2 protein or ACE knockout resulted in improvement of severe lung failure as measured by lung elastance, edema formation, and histological changes associated with acute lung injury (ALI) (Imai et al., 2005). Administration of a single dose of recombinant human ACE2 (rhACE2) to PAH patients resulted in improvement of hemodynamic markers of PAH, including cardiac output and PVR (Hemnes et al., 2018). However, no improvement in clinical or physiological measures of ARDS were seen with the addition of exogenous ACE2 in patients with ARDS (Khan et al., 2017); however, these studies did not have the sufficient statistical power to identify potential clinical benefits. In other models of lung diseases, including bleomycin-induced lung fibrosis and moncrotaline-induced PH, it has been demonstrated that ACE2 plays a critical protective role (Ferreira and Santos, 2005; Yamazato et al., 2009; Zhang et al., 2009). The aforementioned findings support the conclusion that increasing ACE2 expression may present a novel approach for ARDS treatment and emphasize the need for a better understanding of the role of RAS in the pathogenesis of ARDS.

In contrast to the results of experimental ACE2 deletion, ACE knockout and AT1Ra-deficient mice showed marked improvement in ALI symptoms (Imai et al., 2005). Similarly, treatment of chronically hypoxic rats and mice with an AngII receptor antagonist or ACE blocker inhibited pulmonary vascular remodeling associated with the pathogenesis of PH (Nong et al., 1996). ACE-deficient mice with 34% of ACE activity exhibit the same remodeling as normal mice to their pulmonary arterioles, associating the presence of ACE with vascular remodeling (Suylen et al., 2001). In LPS and acid-induced ARDS with high-stretch ventilation, treatment with Ang1–7 reduced the acute inflammatory response and subsequent fibrosis and improved oxygenation (Asperen et al., 2011; Zambelli et al., 2015). Collectively, these studies implicate ACE in the pathogenesis of ARDS (Jerng et al., 2006).

**Figure 1:**
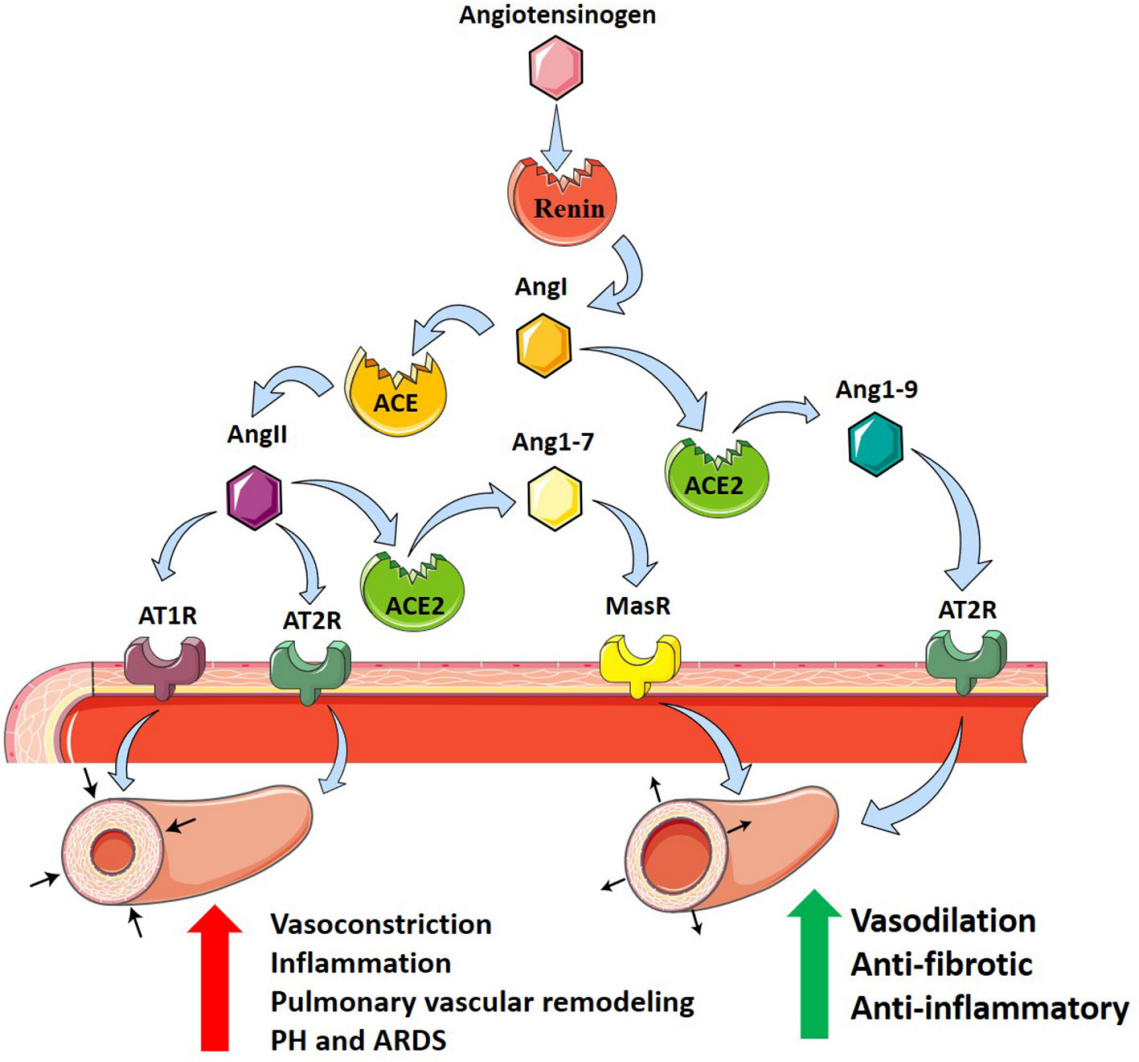
The renin–angiotensin system and its contribution to PH in ARDS. Angiotensinogen (pink hexagon) is cleaved by renin (red circle) into angiotensin I (AngI, orange hexagon). Angiotensin-converting enzyme (ACE, orange circle) cleaves AngI producing angiotensin II (AngII, purple hexagon) which binds to angiotensin II receptor type 1 (AT1R, purple receptor) and angiotensin II receptor type 2 (AT2R, teal receptor), resulting in vasoconstriction, inflammation, and pulmonary vascular remodeling promoting PH in ARDS. Alternatively, angiotensin-converting enzyme 2 (ACE2, green circle) cleaves AngI producing angiotensin1–9 (Ang1–9, blue hexagon) which binds to AT2R, or AngII to produce angiotensin1–7 (Ang1–7, yellow hexagon) which binds to Mas receptor (MasR, yellow receptor). ACE2 counterbalances ACE, protecting against PH in ARDS through anti-fibrotic and anti-inflammatory actions and vasodilation.

In addition to its regulatory role, ACE2 acts as a functional receptor for SARS-CoV (Hamming et al., 2004). SARS-CoV-2 binds to the membrane-bound form of ACE2 leading to the host cell's internalization of the complex (South et al., 2020). Through this mechanism, SARS-CoV-2 and SARS-CoV-1 use ACE2 as a co-receptor for acquiring intracellular entry into the brain and lungs (Kuba et al., 2005; Hoffmann et al., 2020; Wrapp et al., 2020). This internalization of ACE2 results in a decrease of cell surface ACE2, preventing the degradation of disease-promoting AngII and generation of protective Ang1–7 (South et al., 2020). This reduction in pulmonary ACE2 may exacerbate systemic hypertension and PH, fibrosis post-viral infection, and respiratory distress (Imai et al., 2005; Kuba et al., 2005). Potential treatment of COVID-19, with the understanding of the role of ACE2 as a receptor for SARS-CoV-2, could include inhibition of the ACE2 receptor, spike protein-based vaccine, inhibition of transmembrane protease activity, or administration of soluble ACE2 (Cheng et al., 2020). Taken together, these findings support the role of RAS as an important modulator of PH in ARDS where it can modulate both vasoconstriction and vascular remodeling responses (Figure 1).

### The Inflammatory Cascade

Protective mechanical ventilation strategies have improved outcomes of ARDS and PH together, but pulmonary vasculature dysfunction remains in 25% of affected patients and has been increasingly associated with sepsis (Bull et al., 2010; Boissier et al., 2013). Increased flow and ventilatory pressures result in elevated cytokines, notably IL-6, which is attenuated with decreased flow (Ranieri et al., 1999; Samary et al., 2015). This is further highlighted by Pandolfi et al. who examined if IL-6 and acid sphingomyelinase (aSMase) contribute to PH in ARDS (Pandolfi et al., 2017). Through an LPS model of ARDS, they identified that the production of ceramide and IL-6 in rat PASMCs resulted in failed hypoxic vasoconstriction (HPV), endothelial dysfunction, and hyperresponsiveness to pulmonary vasoconstriction induced by serotonin (Pandolfi et al., 2017). Interestingly, blockade of aSMAse attenuated the progression of PH (Pandolfi et al., 2017). Taken together, it is evident that the upregulation of the inflammatory response after lung injury is a central mechanism in the development of PH in ARDS (Price and Wort, 2017).

There exist a multitude of cytokines that have been implicated in the inflammatory cascade in ALI including interleukins (ILs), interferons (IFNs), tumor necrosis factors (TNFs), chemokines, and colony-stimulating factors (CSFs) (Tisoncik et al., 2012). Many of the cytokines associated with the inflammatory cascade are also implicated in the pathogenesis of PH. The number of macrophages significantly increases in plexiform lesions of patients with severe PAH due to increased expression of chemokines in the lungs and chemokine receptors on monocytes (Gerasimovskaya et al., 2012; Florentin et al., 2018). Once activated, macrophages then induce the release of IL-6, IL-10, IL-1β, and TNF-α (Stow et al., 2009). This review will focus largely on IL-6, IL-10, and TNF-α.

IL-6 is an acute phase reactant that is produced as part of an inflammatory response perpetuated by the liver (Heinrich et al., 1990). Serum studies of patients with PAH show increased levels of IL-6 compared with healthy patients, and even demonstrate IL-6 levels as a more accurate prognostic marker than traditional clinical tests (Selimovic et al., 2009; Soon et al., 2010). IL-6 overexpression in mice led to increased muscularization of the proximal arterial tree and distal arteriolar vessels (Savale et al., 2009; Steiner et al., 2009). The muscularization presented with occlusive neointimal angioproliferative lesions that contained primarily endothelial and T cells (Savale et al., 2009; Steiner et al., 2009). This vascular remodeling resulted in an elevated right ventricular systolic pressure and increased right ventricular hypertrophy (Savale et al., 2009; Steiner et al., 2009). Furthermore, IL-6 has also been shown to be involved in the post-transcriptional mechanism of bone morphogenic protein receptor type 2 (BMPR2) downregulation through signal transducer and activator of transcription 3 (STAT3) and the microRNA cluster 17/92 (Brock et al., 2009). IL-6 has also been shown to participate in vascular remodeling by increasing the levels of matrix metallopeptidase 9 (MMP-9) and vascular endothelial growth factor receptor 2 (VEGFR2) that result in the proliferation of PASMCs and trigger the transdifferentiation of pulmonary endothelial cells to PASMCs (Steiner et al., 2009). Most studies have demonstrated that IL-6 exerts a protective effect in ARDS (Groth et al., 2014). This could suggest that the defensive IL-6 response in ARDS can inadvertently result in the development of PH. There are currently multiple clinical trials attempting to elucidate the role of anti-IL6 therapies to attenuate ARDS in COVID-19 (clinicaltrials.gov: NCT 00531856; NCT04363853; NCT04335071). Tocilizumab was used to attenuate the cytokine storm in select patients worldwide, but large randomized controlled trials are yet to show efficacy (Xu et al., 2020).

TNF-α is a pro-inflammatory cytokine secreted by macrophages that regulates cellular proliferation, differentiation, apoptosis, and survival (Parameswaran and Patial, 2010). Increased levels of TNF-α were found in patients suffering from PAH (Soon et al., 2010). TNF-α overexpression in experimental models results in the suppression of prostacyclin (PGI2: a potent vasodilator) mRNA and increased vascular reactivity (Stevens et al., 1992; Itoh et al., 2003). In addition, TNF-α overexpression in type II AECs led to increased septal destruction, bronchiolitis, pulmonary inflammation, and PH (Fujita et al., 2001). An association has been found between polymorphisms in the surfactant protein-B (SP-B) gene and an increased risk of developing ARDS (Gong et al., 2004). TNF expression has demonstrated potent inhibitory activity of SP-B in a human lung adenocarcinoma cell line and decreased surfactant protein-A (SP-A) expression in lung epithelial cells through the p38/MAPK pathway (Berhane et al., 2000; Miakotina and Snyder, 2002). Therefore, this could imply that TNF-α upregulation in ARDS can co-present with PH by inducing atelectasis of alveoli through decreased surfactant production.

IL-10 is an important anti-inflammatory cytokine released by monocytes, macrophages, and several subsets of T cells including type 1 T helper (Th1) cells and regulatory T (Treg) cells (Sabat et al., 2010). Patients with PAH have elevated levels of IL-10, but interestingly increased levels of IL-10 were also observed in patients receiving PGI2 for the treatment of PAH (Groth et al., 2014). In a monocrotaline model of PAH in rats, the intramuscular injection of IL-10 prevented the development of PAH (Ito et al., 2007). Moreover, increased IL-10 production has been associated with the IL-10 promoter polymorphism at position −1082 (−1082GG) (Gong et al., 2006). In a study of 211 Caucasian ARDS patients, the −1082GG genotype resulted in decreased mortality and organ failure in comparison with controls (Gong et al., 2006). It is clear that IL-10 serves a protective role in ARDS and PH; to a greater extent in the −1082GG genotype. However, its efforts may be superseded by a more potent pro-inflammatory response despite persistently elevated IL-10.

Chemokines, or chemotactic cytokines, are a family of 40 low molecular weight proteins that stimulate leukocyte migration and function through their interactions with chemokine receptors (Luster, 1998; Mamazhakypov et al., 2021). There is an elevation in circulating chemokine levels in patients with varying forms of PH which include PH due to chronic obstructive pulmonary disease and lung fibrosis, PAH due to systemic sclerosis, idiopathic PAH, and chronic thromboembolic pulmonary hypertension (CTEPH) (Mamazhakypov et al., 2021). Apart from their roles as biomarkers and in inflammation, chemokines, and their receptors are also involved in pulmonary vascular dysfunction that contributes to PAH (Mamazhakypov et al., 2021). The CCL5–CCR5 axis has been implicated in PAH, most importantly as a mediator of PASMC proliferation *in vitro* (Amsellem et al., 2014). When the CCR5 antagonist maraviroc was administered, the proliferation was depleted (Amsellem et al., 2014). Moreover, CXCL10/CXCL4–CXCR3 axis dysregulation in pulmonary artery endothelial cells led to the loss of recanalization of blocked vessels in CTEPH and impaired angiogenesis (Zabini et al., 2012). CCL3 has also been shown to increase endothelin-1 expression in endothelial cells (Molet et al., 2000). In ARDS, chemokines are released by alveolar macrophages in response to bacterial products (Puneet et al., 2005). However, they can also be secreted by other cells in the local environment as part of an inflammatory cascade involving TNF and IL-1β (Puneet et al., 2005). This demonstrates that an initial inflammatory response in ARDS can be strengthened by an inflammatory cascade and potentiate pulmonary vascular dysfunction and eventual PH.

The importance of the inflammatory cascade in the pathogenesis of PH in ARDS is still not fully understood. Here, we present that a heightened pro-inflammatory response in ARDS can contribute to the development of PH through the involvement of TNF, IL-6, chemokines, and IL-10. These cytokines are implicated in a multitude of processes namely HPV failure, decreased surfactant production, imbalance of vasodilators and vasoconstrictors, PASMC proliferation, and loss of recanalization in blocked vessels. Conclusively, this can provide a new route for effective therapeutics.

### Thrombosis

Thrombosis is a well-described phenomenon that presents in patients suffering from ARDS (Ryan et al., 2014). In a postmortem study of 22 ARDS patients, 19 patients (86%) exhibited macrothrombi in pulmonary arterial and capillary vessels and evidence of microthrombi (Tomashefski et al., 1983). This results from localized damage to the endothelial cell lining that causes the release of tissue factor (TF) in response to inflammation (Maniatis et al., 2008; Levi and Van Der Poll, 2010; Price et al., 2012). TF is a potent activator of the extrinsic coagulation cascade, and experimental models show inhibition of TF–Factor VIIa–Factor X complex reduces the extent of PH in ARDS (Welty-Wolf et al., 2001; MacKman, 2009). Furthermore, plasminogen activator inhibitor-I (PAI-1) levels are elevated in contrast with decreased levels of protein C and thrombin antithrombin (TAT) complexes in ARDS patients (Ware et al., 2003, 2007). These markers suggest that ARDS promotes a hypercoagulable state while repressing fibrinolysis and is also associated with increased mortality (Ware et al., 2007).

Interactions between coagulation and an excessive inflammatory response are considered essential in the pathophysiology of ARDS (Frantzeskaki et al., 2017). Activation of coagulation pathways in ARDS results in the overproduction of thrombin that can further ameliorate inflammatory processes through proteinase-activated receptors (Frantzeskaki et al., 2017). Under non-pathological conditions, thrombin production is highly regulated by tissue factor pathway inhibitor, anti-thrombin III, and the protein C system (José et al., 2014). These feedback mechanisms can be diminished in inflammation due to increased consumption and decreased production of anticoagulants (José et al., 2014). It is evident that the formation of microthrombi is due to increased coagulability and can promote PH in ARDS patients through the pathological blockade of vessels.

**Figure 2:**
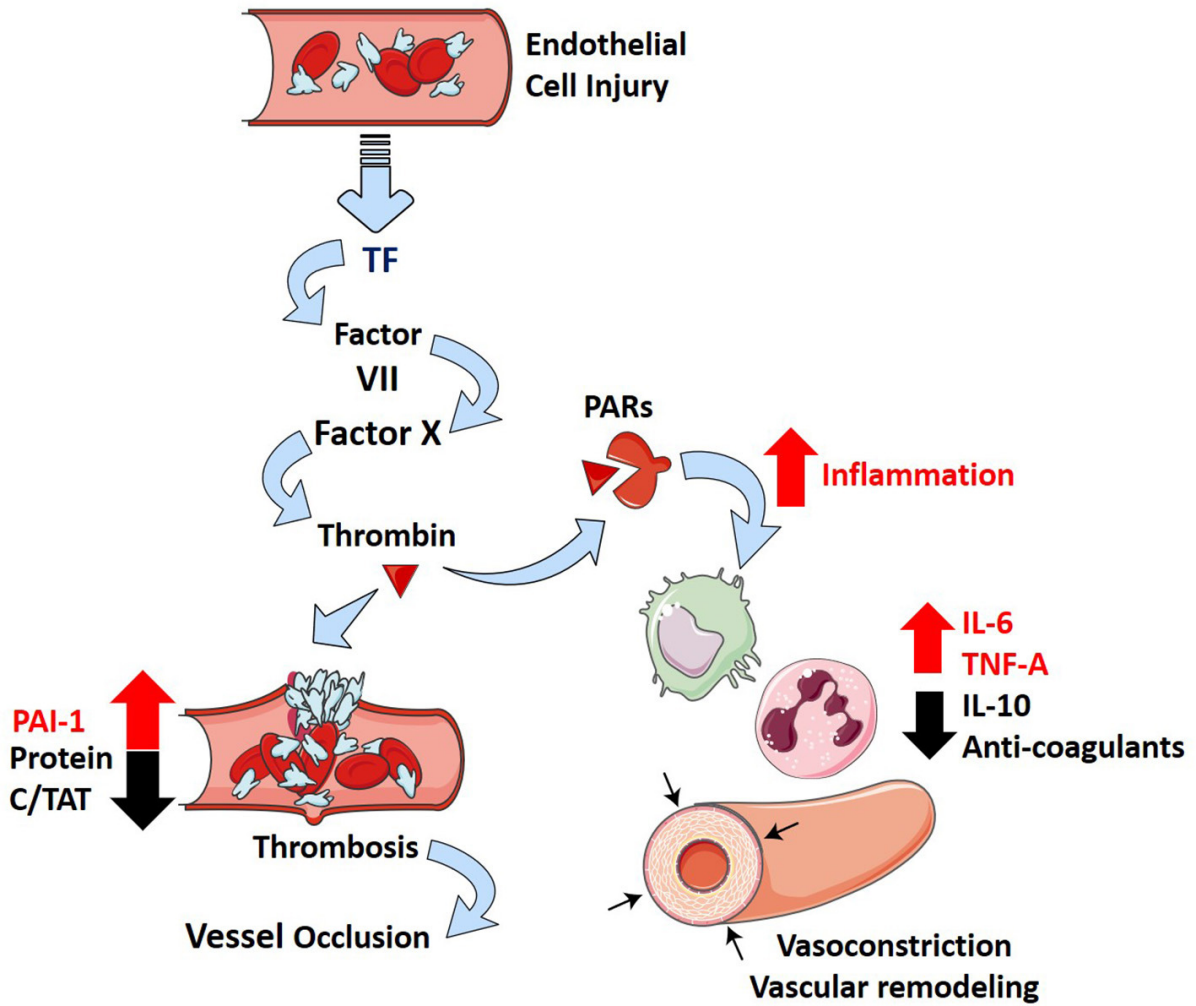
Inflammation and thrombosis in ARDS and how they contribute to PH. Injury to the alveolar-capillary interface in ARDS leads to the release of tissue factor from the endothelial cells and induces coagulation through the extrinsic pathway. The penultimate mediator of coagulation, thrombin (dark red triangle), interacts with proteinase-associated receptors (PARs, dark red receptor) to promote inflammation (increase in IL-6, TNF-α, and IL-10) and suppress several negative regulatory elements of coagulation (decrease in Protein C, thrombin antithrombin complexes—TATs, anticoagulants; increase in plasminogen activator inhibitor 1—PAI-1). This leads to downstream effects of inflammatory cytokines (IL-6 and TNF-α) that include vascular remodeling and vasoconstriction, which overwhelm the protective effects of anti-inflammatory cytokine IL-10 and further incite thrombosis.

More recently, ARDS secondary to SARS-CoV-2 infection (COVID-19) has been associated with an increase in the risk of thrombotic complications (mainly pulmonary embolisms) in comparison with non-ARDS SARS-CoV-2 infections (Helms et al., 2020). Longer prothrombin time (PT), increased levels of D-dimer and fibrin degradation products, longer activated partial thromboplastin time (aPTT), and decreased platelets were found in non-survivors of SARS-CoV-2 (Tang et al., 2020b; Xiaofang et al., 2020). These parameters are all related to a poor prognosis in infected patients (Tang et al., 2020b; Xiaofang et al., 2020). While experimental models of SARS-CoV-2 infections have not been widely studied, infection of SARS-CoV with ARDS results in increased fibrin deposition in mice (Gralinski et al., 2013). Furthermore, the use of low molecular weight heparin leads to reduced mortality in severe COVID-19 patients (Tang et al., 2020a). This suggests that a similar balance toward coagulation might exist in patients with ARDS-related SARS-CoV-2 and might provide significant prognostic value in severe infections. How the inflammatory and thrombotic pathways contribute to PH are represented diagrammatically in Figure 2.

### Hypoxic Pulmonary Vasoconstriction

HPV is a unique and complex feature of the pulmonary circulation whereby hypoxic conditions lead to vasoconstriction, in contrast to the systemic circulation where hypoxia leads to vasodilation (Lumb and Slinger, 2015). HPV has an important physiological role; it acts to match perfusion to ventilation to optimize P_O2_ (McLoughlin, 2018). Thus, HPV shunts blood toward areas with high ventilation to maintain efficient gas exchange processes (Sylvester et al., 2012). In ALI settings, both clinically and experimentally, edema as a result of loss of barrier function leads to regional hypoxemia, activating HPV to shunt blood away from hypoxic areas of the lung to ventilated areas (Marshall et al., 1994; Fischer et al., 2004; Spöhr et al., 2007; Sylvester et al., 2012). This beneficial effect of HPV has been shown to be inhibited in experimental models of oleic acid, bleomycin, or endotoxin-induced ALI (Sylvester et al., 2012), underscoring the important role that HPV has in matching ventilation/perfusion. Herein, studies in mice have revealed that inhibition of ATP-regulated potassium channel Kir6.1, which is elevated after endotoxemia, was able to restore HPV in mice; however, the effects on lung inflammation were not assessed (Turzo et al., 2018). However, prolonged HPV can lead to the development of PH and high-altitude induced pulmonary edema (HAPE) which is characterized by patchy peripheral distribution of edema (Bartsch, 1999). Although this mechanism is distinct to the classic presentation of ARDS that has a strong inflammatory component, HAPE is characterized by the presence of pulmonary edema (Bartsch, 1999). Pulmonary edema is also a feature shared with ARDS that contributes to deficits in gas exchange and lung failure due to fluid accumulation in the alveolar spaces (The ARDS Definition Task Force, 2012). Thus, although distinct to ARDS, HAPE as a result of prolonged HPV represents a mechanism linking PH and the development of edema, a feature of ARDS.

An important downstream molecular consequence of hypoxia is the stabilization of hypoxia-inducible factor (HIF)-1A and HIF-2A. Under normoxic conditions, HIFs are degraded by prolylhydroxylases (PHDs); however, under hypoxia, the PHDs are inactivated allowing HIFs to stabilize (Eltzschig and Carmeliet, 2011). In ALI, the stabilization of HIF1A and HIF2A have been shown to limit injury (Karmouty-Quintana et al., 2013b; Gong et al., 2015; Huang et al., 2019). Deletion of endothelial cell HIF-1A expression inhibited endothelial proliferation and attenuated vascular repair processes in a model of sepsis-induced lung injury (Huang et al., 2019). Similarly, abrogation of endothelial HIF-2A resulted in defective adherens junction that was exacerbated after endotoxin challenge in mice, resulting in worsened lung injury (Gong et al., 2015). The downstream HIF-mediated mechanism is complex and includes many mediators; however, increased expression of ecto-nucleotidases CD39 and CD73 and the increased expression of the adenosine A2B receptor (ADORA2B) have been implicated in the accumulation of adenosine and subsequent protective effects of adenosine through activation of its receptors (Karmouty-Quintana et al., 2013b). Remarkably, these same mechanisms that are protective in acute lung injury settings have also been linked with the pathophysiology of PH. Genetic deletion of endothelial PHD2 and subsequent stabilization of HIF-2A has been linked with the spontaneous development of PH in mice associated with severe vascular remodeling (Dai Zhiyu et al., 2016). Several studies have also demonstrated an important role for HIF1A, adenosine, and ADORA2B activation as causative mediators in group 3 PH (Karmouty-Quintana et al., 2012, 2013a; Garcia-Morales et al., 2015; Rajagopal et al., 2021). Taken together, these studies illustrate the complexity of hypoxic–adenosinergic responses in the pathophysiology of ARDS and PH, whereas they are protective in ALI settings, they also promote features of PH and chronic lung injury. This duality is perhaps best highlighted by studies that determined the prevalence of HIF2A and PHD2 polymorphisms that are associated with improved adaptation to hypoxia in high-altitude residents in patients with ARDS. This study demonstrated that the PHD2 polymorphism *PHD2 RS516651-TT*, which is associated with increased adaptation to high altitude, was associated with a higher 30-days mortality risk within 30 days of the onset of ARDS (Dötsch et al., 2017).

### Endothelin

Increased levels of endothelin-1 (ET-1), a potent vasoconstriction agent (Davenport and Maguire, 1994), have been reported clinically in ARDS patients where they contribute not only to pulmonary vasoconstriction but also to promote lung edema (Druml et al., 1993; Sanai et al., 1996; Nakano et al., 2007). These observations are significant because inhibition of ET-1 is an important strategy in the treatment of PH (Clozel et al., 2013). Endothelin receptor antagonists (ERAs) have been used successfully in experimental models of ALI where they inhibited lung edema; unfortunately, pulmonary vascular pressures were not assessed in these studies (Guimarães et al., 2000; Araz et al., 2013). However, due to the effect of ERAs in modulating PH by promoting vasodilation and reducing inflammation in both PH and ALI (Guimarães et al., 2000; Araz et al., 2013; Clozel et al., 2013), its use in ARDS as a front-line therapy has been postulated (Araz, 2020).

### Hyaluronan

**Figure 3:**
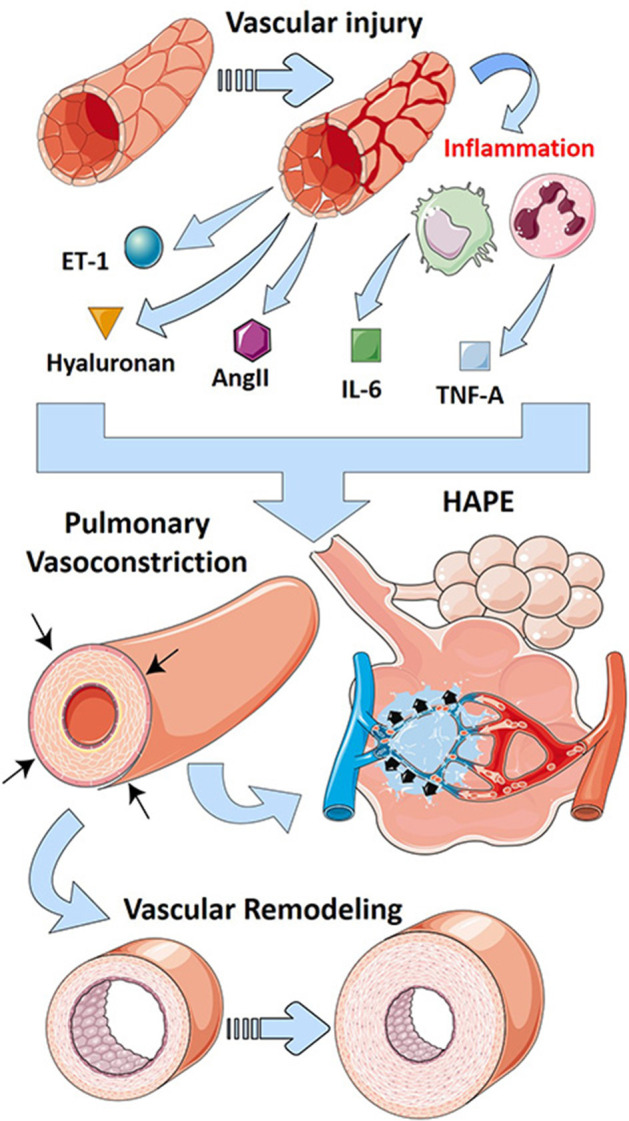
Mechanisms that lead to PH in ARDS. Vascular injury in ARDS leads to the release of vasoactive mediators including endothelin-1 (ET-1-blue/green sphere), hyaluronan (golden triangle), and angiotensin II (AngII, purple hexagon). Further, vascular injury promotes inflammation that releases cytokines such as interleukin-6 (IL-6, green squares) and tumor necrosis factor (TNF)-α (blue squares). These vasoactive mediators and cytokines promote vascular remodeling and pulmonary vasoconstriction, hallmarks of pulmonary hypertension. Prolonged vasoconstriction can then lead to high-altitude pulmonary edema (HAPE), a form of lung injury similar to ARDS.

PH in the setting of ARDS falls into WHO group 3 PH because it is strongly associated with hypoxia and the presence of lung injury (Poor et al., 2012). A recent review of mechanisms of group 3 PH associated with lung fibrosis has been recently published (Rajagopal et al., 2021). Interestingly, similar pathways are involved in both PH associated with ARDS and lung fibrosis such as inflammatory mediators, RAS, and endothelins (Rajagopal et al., 2021). In addition, the glycosaminoglycan hyaluronan has been implicated in PH associated with chronic lung diseases (Collum et al., 2017, 2019). Interestingly, hyaluronan is elevated in experimental lung injury (Ni et al., 2018; Bell et al., 2019), and its inhibition has been shown to be effective in models of ALI (McKallip et al., 2013, 2015). Thus, it is conceivable that in addition to promoting lung inflammation, excess hyaluronan in ARDS may also contribute to PH. In line with this, we hypothesize that the hypoxic environment in ARDS leads to increased expression or activity of CD73 and ADORA2B such as in PH in the setting of lung fibrosis (Garcia-Morales et al., 2015) that leads to accumulation of adenosine and subsequent activation of ADORA2B. Activation of this receptor has been shown to promote hyaluronan production through increased expression of hyaluronan synthases (HAS)-1 and 2 (Karmouty-Quintana et al., 2013a; Mertens et al., 2018). Interestingly, many recent publications have linked increased hyaluronan deposition in COVID-19 (Hellman et al., 2020; Kaber et al., 2020), a disease that is more and more associated with important cardiopulmonary complications (Karmouty-Quintana et al., 2020; Potus et al., 2020). Recent publications from our group have also demonstrated that inhibition of hyaluronan by 4-methylumbelliferone (4MU) attenuated PH by diminishing vascular remodeling (Collum et al., 2017, 2019). 4MU has also been postulated as a therapeutic for COVID-19–induced ARDS where it could not only attenuate the inflammatory effects but also dampen the cardiovascular complications (Karmouty-Quintana et al., 2020; Shi et al., 2020). The main pathways that contribute to PH after vascular injury are summarized in Figure 3.

## Conclusions

Mechanisms of PH in ARDS are complex and poorly understood. Evidence that PH increases the mortality in patients with ARDS is suggested by some studies, but definitive evidence is thwarted by inconsistent reports (Beiderlinden et al., 2006; Ñamendys-Silva et al., 2014; Calcaianu et al., 2017). However, it is clear that pulmonary vascular dysfunction is present in patients with ARDS and has indirect associations with mortality (Monchi et al., 1998; Bull et al., 2010). This review summarizes current understanding of the pathways that contribute to PH in ARDS, with specific commentary regarding the implications for the current COVID-19 pandemic.

The renin–angiotensin axis plays an important and multi-dimensional role in pulmonary vascular changes in both acute and chronic lung disease (Jia, 2016). A hyperactive form of ACE correlates with the development of and increased mortality in ARDS (Marshall et al., 2002; Jerng et al., 2006). Further, the increases in ACE and its specific catabolites have been shown to directly contribute to vascular remodeling in PH (Krege et al., 1995; Nong et al., 1996; Suylen et al., 2001). For example, activation of the AngII receptor AT1R directly promotes vasoconstriction and vascular remodeling, prominent drivers of PH (Iwai and Horiuchi, 2009). Conversely, the ACE homolog ACE2 is protective in PH, and increasing its expression would be a novel approach to PH-ARDS treatment (Imai et al., 2005). It is not surprising then that the loss of membrane-associated ACE2 in the lung caused by SARS-CoV has been proposed as a crucial mechanism for the rapidly progressive alveolar damage and vascular changes present in COVID-19 patients (Imai et al., 2005; Kuba et al., 2005). There are multiple clinical trials in the works investigating the prophylactic and therapeutic effect of angiotensin pathway–modulating drugs in preventing and treating patients with COVID-19–associated ARDS (clinicaltrials.gov; NCT04335786; NCT04337190).

Another critical contributing mechanism to ALI is a robust and exaggerated immune response, referred to here as the inflammatory cascade. Acute inflammation characterized by rapid and robust release of inflammatory cytokines such as IL-6, IL-10, and TNF is a central feature of ARDS (Tisoncik et al., 2012). These same mediators are also implicated in PH and may play key roles in the severe and acute inflammation observed in ARDS with PH. Of particular interest, the destructive IL-6 response in ARDS may contribute to the subsequent observations of PH in ARDS patients (Groth et al., 2014; Pandolfi et al., 2017). Therapeutics which target these inflammatory pathways are worth serious investigation. The inflammatory cascade, as a component of cytokine release syndrome, is thought to be involved in the development of ARDS in patients infected with SARS-CoV-2, where important changes to the pulmonary vasculature including features of PH have been reported (Potus et al., 2020; Sun et al., 2020). There are currently randomized controlled trials evaluating the efficacy of leronlimab, a CC chemokine receptor 5 (CCR5) modulator, in patients with mild to severe SARS-CoV-2–induced ARDS (clinicaltrials.gov, NCT04347239).

The ARDS-related inflammatory cascade has also been tied directly to another feature of ARDS: hypercoagulability and thrombosis. An altered thrombotic and fibrinolysis profile has been linked to PH in ARDS (Nieuwenhuizen et al., 2009). Inhibition of the coagulation cascade abrogates ARDS-related PH (Welty-Wolf et al., 2001; MacKman, 2009). In this review, we describe several molecular mechanisms that perpetuate this abnormal hemostatic state, and provide insight into novel diagnostic and therapeutic interventions that can increase positive outcomes in ARDS. The presence of underlying viral infections, in particular COVID-19 infection, in ARDS patients even further increases the risk of thrombotic events. While dysregulation of the coagulation cascade is observed in COVID-19 patients with either or both ARDS and PH, the mechanisms by which this occurs are not fully understood, thwarting the informed use of agents which interfere with hypercoagulability in these patients.

The unique vasoconstrictive response of the lungs to hypoxia presents a particularly complex phenomenon in ARDS and PH (Lumb and Slinger, 2015). Acute pulmonary edema in ALI activates the hypoxic response, which allows the lungs to maximize oxygenation efficiency (Marshall et al., 1994; Fischer et al., 2004; Spöhr et al., 2007; Sylvester et al., 2012). However, if HPV becomes chronic, it can result in the development of PH (Bartsch, 1999). As such, potential therapeutic enhancement of HPV in acute settings must be balanced by consideration of the long-term effects of HPV, which may increase the risk of PH. Viral infections are known to lead to hypoxia through pulmonary vascular and epithelial cell damage (Sun et al., 2020). As such, the role of disrupted vascular regulation in patients with severe COVID-19 warrants further investigation.

Two additional phenomena observed in ARDS are increased circulating ET-1 levels (Nakano et al., 2007) and exacerbated local deposition of hyaluronan in the lung parenchyma (McKallip et al., 2013, 2015; Ni et al., 2018; Bell et al., 2019). Interestingly, inhibition of both ET-1–mediated actions (Guimarães et al., 2000; Araz et al., 2013) and hyaluronan deposition (McKallip et al., 2013, 2015) are beneficial in models of ALI. While hyaluronan has been implicated in the pathogenesis of PH related to chronic lung disease (Collum et al., 2019; World Health Organization, 2020), neither ET-1 nor hyaluronan has been thoroughly investigated in ARDS-related PH.

An important aspect in the pathophysiology of PH in ARDS is the interplay between acute and chronic mediators that may promote features of PH. In the early stages of ALI, damage to the endothelium and subsequent increase of vasoactive mediators such as ET-1, IL-6, and TNF-A can promote pulmonary vasoconstriction leading to increased PVR. Similarly, loss of ACE2, such as after viral infections, leading to increased AngII and subsequent activation of AT2 receptors can also promote pulmonary vasoconstrictor responses. An important aspect of PH is vascular remodeling. Although it is unclear at what stage vascular remodeling is initiated in ARDS, it is conceivable that the protracted presence of mediators such as HIFs and the accumulation of adenosine initiate vascular remodeling processes that contribute further to the pathogenesis of PH in ARDS. In line with this, chronic phase of ARDS is known for its fibroproliferative milieu, where HIF (Delbrel et al., 2018), adenosine (Karmouty-Quintana et al., 2013b), and hyaluronan (Li et al., 2011) play central roles.

Given the ongoing COVID-19 global pandemic, it is crucial for the authors to address the implications of the aforementioned findings for patients with COVID-19. COVID-19 has infected over 64 million individuals and resulted in over 1.3 million deaths worldwide (https://coronavirus.jhu.edu/map.html accessed December 2, 2020). ARDS is the most common clinical course for patients with severe COVID-19 infections (Machhi et al., 2020). Prognosis for patients with ARDS is dismal, and some recent evidence suggests that the patients with COVID-19–related ARDS may face an even higher risk of death. As effective treatments for ARDS are lacking and a vaccine against COVID-19 has yet to be developed, it is critical that targetable mechanisms behind this devastating disease are discovered. A recent review has summarized the known mechanisms to date that promote pulmonary vascular dysfunction in SARS-CoV-2–induced ARDS and identified novel potential treatment options that target the vasculature (Karmouty-Quintana et al., 2020).

## Author Contributions

LR and AH contributed equally. All authors read and approved the manuscript.

## Conflict of Interest

The authors declare that the research was conducted in the absence of any commercial or financial relationships that could be construed as a potential conflict of interest.
